# Implementation of an educational intervention to improve hand washing in primary schools: process evaluation within a randomised controlled trial

**DOI:** 10.1186/1471-2458-13-757

**Published:** 2013-08-15

**Authors:** Catherine R Chittleborough, Alexandra L Nicholson, Elaine Young, Sarah Bell, Rona Campbell

**Affiliations:** 1 School of Social and Community Medicine, University of Bristol, 39 Whatley Road, Bristol BS8 2PS, United Kingdom; 2 School of Population Health, University of Adelaide, 178 North Terrace, South Australia 5005, Australia

**Keywords:** Hand washing, Primary school, Process evaluation, Educational intervention

## Abstract

**Background:**

Process evaluations are useful for understanding how interventions are implemented in trial settings. This is important for interpreting main trial results and indicating how the intervention might function beyond the trial. The purpose of this study was to examine the reach, dose, fidelity, acceptability, and sustainability of the implementation of an educational hand washing intervention in primary schools, and to explore views regarding acceptability and sustainability of the intervention.

**Methods:**

Process evaluation within a cluster randomised controlled trial, including focus groups with pupils aged 6 to 11, semi-structured interviews with teachers and external staff who coordinated the intervention delivery, and school reports and direct observations of the intervention delivery.

**Results:**

The educational package was delivered in 61.4% of schools (85.2% of intervention schools, 37.8% of control schools following completion of the trial). Teachers and pupils reacted positively to the intervention, although concerns were raised about the age-appropriateness of the resources. Teachers adapted the resources to suit their school setting and pupils. Staff coordinating the intervention delivery had limited capacity to follow up and respond to schools.

**Conclusions:**

The hand washing intervention was acceptable to schools, but its reach outside of a randomised trial, evidenced in the low proportion of schools in the control arm who received it after the trial had ended, suggests that the model of delivery may not be sustainable.

**Trial registration:**

ISRCTN: ISRCTN93576146

## Background

Understanding how interventions are implemented in trial settings is important for interpreting the main trial results and indicating how the intervention might function beyond the trial [1,2]. Process evaluation can assess where, when, and why variations in implementation occur [3]. Variations can be measured by the degree to which the intervention was conducted as planned (fidelity), including adherence to protocol, quality of delivery, and participant response (acceptability) [4,5]. The amount of intervention delivered (dose), and proportion of intended target population receiving it (reach) can also be used to assess implementation [1,6]. Such data can shed light on why an intervention may or may not be effective and indicate how sustainable the delivery model might be if rolled out into routine practice.

The ‘Hands up for Max!’ hand hygiene study is a cluster randomised controlled trial (RCT) to determine whether an educational package to promote hand washing is effective in reducing absenteeism among pupils and staff in primary schools. Hand hygiene is important in preventing infection as person-to-person contact, including via hands, is a common mode of transmission for gastrointestinal and respiratory infections [7]. Previous educational interventions in school settings have been associated with reduced absenteeism [8,9] and gastrointestinal infections [10], and increased compliance with hand washing [11,12]. Reviews of interventions to promote hand washing, including several studies in school settings, concluded that the interventions were associated with reduced respiratory infections, but that the studies were generally poor quality [13,14]. Interventions to improve water quality, hygiene behaviours and sanitation in schools have also been shown to reduce absence among primary school pupils in developing countries [15].

If results from the trial of the ‘Hands up for Max!’ intervention indicate that it is effective in reducing absenteeism, successful transfer of the intervention into school practice may depend on many factors not evident in quantitative analyses of the main trial data [1]. Conversely, negative results may be due to implementation not occurring as intended [16]. This paper uses process evaluation data embedded within a cluster RCT to determine the fidelity, dose and reach of this intervention, and to consider issues of acceptability and sustainability beyond the trial setting.

## Methods

### ‘Hands up for Max!’ hand hygiene resource

The ‘Hands up for Max!’ educational resource pack was developed, formatively evaluated and refined by the former Health Protection Agency (HPA) in England (now part of Public Health England) as a low cost educational intervention easily integrated into existing school curricula ( http://www.hpa.org.uk/Topics/InfectiousDiseases/InfectionsAZ/Handwashing/handwHandwashinginprimaryschoolsresources/). The final version used in the trial included a five-minute CD-ROM or DVD animation teaching how to wash hands correctly, lesson plans exploring ‘What are germs?’ and ‘Healthy hands, healthy school’ , A4 posters demonstrating how to wash hands correctly, and stickers for pupils. The pack also included several optional resources including fun facts (background information), homework activities, a Max hand washing game, and an art competition that could be used alongside lesson plans. For implementation of the intervention, schools were expected to deliver at least the lesson plans and the animation demonstrating how to wash hands correctly. University or HPA staff went through the resources in the intervention pack during a pre-arranged telephone call with the teacher responsible for delivery of the intervention. Teachers were also provided with guidance on planning the delivery of the intervention in their school, including disseminating intervention materials to other staff in the school, photocopying lesson plans and worksheets, and gathering or purchasing glitter, soap, paper towels and buckets required for the lessons.

### Sample

All state primary schools within six Local Authorities in the South West of England were invited to participate in the trial. Participating schools (n=178) were randomised to receive the ‘Hands up for Max!’ educational package in October 2009 (intervention schools, n=88) or to receive the resource after follow-up data collection for the main trial was completed (control schools, n=90). Resource packs were sent to control schools between September 2010 and March 2011. Routine absence data will be used to assess the primary outcome of the number of half days of pupil absence from school in all 178 schools. Within the main trial, 24 schools (12 intervention and 12 control) were randomly selected to be part of a sub-study in which enhanced data on staff and pupil absenteeism will examine infection-related absence. Schools in the sub-study received £1000 in four instalments during the four months of the sub-study (January to April 2010) to reimburse them for administrative staff costs associated with organisation and collection of enhanced absenteeism data.

### Process evaluation

A range of data sources were used to explore how the intervention was implemented and received (Table 1). These included log sheets detailing communications with schools (n=178 schools), interviews with HPA and university staff coordinating intervention delivery to all schools (n=8 interviews), and direct observation, pupil focus groups and teacher interviews (n=4 intervention schools). Four intervention and four control schools were purposively selected from the sub-study to be involved in the direct observation, pupil focus groups, and teacher interviews of the process evaluation based on the strata of school size (large >194 pupils vs. small) and proportion eligible for free school meals (high >6.4% vs. low) dichotomised at the median. No additional honorarium was provided to these schools, teachers or pupils. Findings from these direct observations, pupil focus groups, and teacher interviews, designed to explore facilities, knowledge, attitudes, and behaviours related to hand washing, have been reported previously [17]. This paper uses data from these observations, focus groups, and teacher interviews related to how the intervention was implemented in these four intervention schools. University researchers arranged appointments with schools for the qualitative data collection to be conducted. Two or three separate visits were required to conduct the focus groups, interviews, and direct observations in intervention schools. The focus groups and interviews were conducted within two weeks of the intervention being delivered.

**Table 1:** Data sources used to assess implementation of the ‘Hands up for Max!’ educational resource

			**Implementation aspect assessed**				
**Data source**	**Sample**	**Date of data collection**	**Reach**	**Dose**	**Fidelity**	**Acceptability**	**Sustainability**
Log sheets recording communication with schools	178 schools		X	X	X	X	
	88 intervention	Oct 2009-					
	90 control	Dec 2011					
Interviews with staff coordinating intervention delivery	2 HPA, 1 University staff	May 2010			X	X	X
	4 HPA, 1 University staff	Mar-Jun 2012					
Direct observation of intervention delivery	4 intervention schools: Lesson delivery was observed in one KS1 and one KS2 class at each school; Use of the DVD and other intervention components was observed in each of the 4 schools	Nov-Dec 2009		X	X	X	
Pupil focus groups	8 focus groups: 2 focus groups (1 lower and 1 upper KS2 group) at each of the 4 intervention schools, with a total of 49 pupils (19 male, 30 female)	Nov-Dec 2009			X	X	
Interviews with teachers	8 interviews: 2 interviews (1 KS1 and 1 KS2 class teacher) at each of the 4 intervention schools, all female	Nov-Dec 2009			X	X	X

*Note*. *HPA* Health Protection Agency, *KS* Key Stage.

#### Log sheets

Log sheets were used by HPA staff and University staff to record communication about delivery of the resource with schools via telephone and email in the 88 intervention schools and 90 control schools who received the educational package following completion of the trial data collection.

#### Interviews with staff

Eight semi-structured interviews were conducted in 2010 and 2012 with four HPA staff and two University staff involved in coordinating the intervention delivery. Two HPA staff members were interviewed at both time points (Table 1). The staff interviews in 2010 were conducted following delivery of the educational resource in intervention schools, which occurred between October 2009 and May 2010. The interviews in 2012 were conducted following the delivery of the intervention in control schools, which occurred between October 2010 and July 2011. The interviews explored views on delivering the intervention and the factors that enhanced or diminished the likelihood of the intervention being delivered. The mean duration of these eight interviews was 24 minutes (range 17 to 34).

#### Direct observation of intervention delivery

Observation checklists were used to document how the intervention was delivered to Key Stage 1 (KS1, Years 1 to 2, ages 5 to 6) and Key Stage 2 (KS2, Years 3 to 6, ages 7 to 11) in the four selected intervention schools. Checklists included information on the elements of the intervention package that were used, how each was delivered (e.g. in assembly or individual classes), how long lessons took, and how pupils reacted. Observation of the delivery of the intervention usually required the researcher to be present on more than one day. While the researcher did not observe all classes in the school where the educational resource was being used, two or three visits were usually required to ensure that observation captured the various components of the educational resource being used across at least one KS1 and one KS2 class.

#### Focus groups with pupils

Focus groups were conducted with pupils from one lower KS2 class (ages 7 to 9) and one upper KS2 class (ages 9 to 11) in each of the four selected intervention schools, except for one where the younger focus group was conducted with KS1 pupils in Year 2 (ages 6 to 7, Table 1) because this was the class the school had selected to participate. In most classes pupils were randomly selected from those in the class whose parents had consented to them participating. In some classes, pupils participating in the focus group represented all those who had returned their parental consent forms, or were selected by the teacher in which case the basis for selection was unknown. Focus groups were conducted with 5 to 7 pupils and took place during school hours in a variety of settings, including classrooms and libraries, depending on room availability. The mean duration of the focus groups was 37 minutes (range 25 to 52). There is increasing acknowledgement of the validity of children’s own views within research, rather than obtaining children’s perspectives indirectly through the adults who are responsible for them, such as parents, care givers, or teachers [18-20]. Groups comprised children of similar ages because of wide variations in comprehension with small differences in age [21]. It has been previously recommended that children should be 6 years or older to possess the social and language skills required to be effective participants in focus groups [22]. The focus groups were piloted with pupils in Year 1 (age 5), but this younger age group was not included in the process evaluation because of difficulties obtaining responses to questions without significant prompting, and uncertainty about whether the pupils comprehended what they were agreeing to during the assent process. Focus groups explored children’s views on hand washing facilities in the school, their thoughts on barriers and facilitators to good hand washing, and, in intervention schools only, their response to the ‘Hands up for Max!’ intervention. A participatory drawing activity was included to help children express their thoughts about the facilities they use to wash their hands. Results related to views on hand washing and hand washing facilities are reported separately [17]. Questions were kept simple and concise, and were worded to avoid eliciting yes/no answers. A co-facilitator was present at each focus group to assist any child who needed help, and with technical details related to the digital recorder and taking notes to aid transcription.

#### Interviews with teachers

Semi-structured interviews were conducted with two teachers (one KS1, one KS2, Table 1) from each of the four selected intervention schools and explored views on the intervention and how it was delivered. If there was more than one KS1 or KS2 teacher at the school, the staff, sometimes with the head teacher, decided on the teacher that would participate in the interview, mainly based on availability. The mean duration of these interviews was 16 minutes (range 10 to 24).

### Ethics

This study was approved by the University of Bristol Faculty of Medicine and Dentistry Committee for Ethics. A multi-level process of consent was used. Head teachers consented for the schools to be involved in the trial. Parents/guardians gave written consent for their child to be involved in the focus group. Children who participated in the focus groups were provided with a verbal description of the research before being asked to assent. Written consent was also provided by teachers and staff before the interviews.

### Analyses

The proportion of the intended target population in intervention and control schools who actually received the intervention (reach) was determined from information reported in the log sheets. Association of characteristics of schools (number of pupils, proportion of pupils eligible for free school meals, geographic area, involvement in substudy) and delivery of the education program were examined with generalised linear regression models using a binomial distribution and a log link function. Schools were considered to have fully delivered the program if they had used at least the animation and lesson plans across all KS1 and KS2 classes. Schools delivering the resource only to some classes, or if they had not used both the animation and the lesson plans were considered to have partially delivered the intervention. For the analyses examining differences between schools that did and did not deliver the intervention, the definition of having delivered the intervention included both full and partial delivery. A univariable model first examined the association of intervention delivery with each school characteristic separately. A multivariable model then mutually adjusted each school characteristic for all other variables listed in Table 2. Stata version 12 was used for these quantitative analyses.

**Table 2:** Relative risks of school characteristics associated with full or partial delivery, compared to non-delivery, of the education program (n=178 schools)

		**Univariable**		**Adjusted**	
	**%**	**RR (95% CI)**	**p value**	**aRR (95% CI)**	**p value**
**Study allocation**					
Control	37.8	ref		ref	
Intervention	85.2	2.26 (1.71, 2.98)	<0.001	2.21 (1.69, 2.90)	<0.001
**Area**					
Bristol	42.9	ref		ref	
Bath and North East Somerset	94.4	2.20 (1.41, 3.43)	<0.001	2.22 (1.39, 3.56)	0.001
North Somerset	63.6	1.48 (0.90, 2.45)	0.122	1.41 (0.89, 2.27)	0.143
South Gloucestershire	53.9	1.26 (0.72, 2.20)	0.423	1.06 (0.65, 1.74)	0.807
Swindon	60.0	1.40 (0.80, 2.45)	0.238	1.56 (0.89, 2.70)	0.118
Wiltshire	62.3	1.45 (0.90, 2.34)	0.125	1.46 (0.90, 2.35)	0.126
**Involved in substudy**					
No	60.4	ref		ref	
Yes	66.7	1.10 (0.81, 1.51)	0.534	1.43 (1.04, 1.98)	0.030
**Number of pupils**					
194 or less	60.2	ref		ref	
>194	62.4	1.04 (0.82, 1.31)	0.770	1.01 (0.83, 1.24)	0.903
**Proportion eligible for free school meals**					
6.4% or less	65.2	ref		ref	
>6.4%	57.3	0.88 (0.69, 1.11)	0.285	0.88 (0.71, 1.08)	0.219

*Note*. Adjusted model includes all variables in table; *CI* confidence interval, *RR* relative risk, *aRR* adjusted relative risk, *ref* reference category.

Information about the degree to which the intervention was conducted as planned (fidelity), whether the specific components of the intervention were delivered (dose), and the acceptability and sustainability of the education program, was obtained from focus groups with pupils, interviews with teachers and HPA staff, and direct observation of intervention delivery. Atlas.ti was used to aid the qualitative data organisation and analyses. Some information about how the intervention was delivered was recorded in log sheets, although these were not designed specifically to collect data on this issue. The digital recording of each focus group and interview was transcribed verbatim. Each transcript was checked for accuracy by the researcher who conducted the focus group or interview. A conceptual framework was developed to classify and organise data [23], and included topics and subtopics that were deductively derived from issues introduced in the focus group and interviews according to the topic guide. Within these subtopics, codes were created based on recurring accounts identified in the transcripts and checklists. Two authors (AN and EY) independently reviewed transcripts and formulated codes. Codes across all subtopics were inductively sorted into potential themes and relevant data extracts were collated within identified themes [24,25]. Thematic networks were constructed to facilitate the structuring, description, and interpretation of the themes [24]. Case ordering within themes, whereby cases (interviews and focus groups) were ordered according to some variable of interest, for example positively worded statements, through neutral to negatively worded statements, enabled examination of similarities and differences across cases, for example between teachers and HPA staff [26].

## Results

### Reach

Overall, 61.4% of schools delivered the educational package completely (n=101 schools) or partially (n=8). HPA and university staff were unable to determine if it was delivered in 16.9% of schools (27 control and 3 intervention schools). Reasons stated on the log sheets for non-delivery included the school being too busy (6 control schools) and staff changes (3 control, 1 intervention school). One intervention school refused to deliver the program and one control school closed during the study. Reasons for non-delivery were unknown for 19 control and 8 intervention schools because schools did not provide a reason in their phone or email communication with HPA or university staff. Intervention schools were more likely to deliver the program than control schools (adjusted Relative Risk, RR 2.21, 95% confidence interval, CI, 1.69, 2.90, Table 2). The proportion of schools who delivered the educational package (fully or partially) varied by geographic area, the highest proportion being in Bath and North East Somerset. Schools participating in the sub-study were more likely to deliver the program (Table 2).

### Dose

The educational resource reached pupils in 61.4% of schools, but there was variation across and between schools in the dose received by pupils and the fidelity of implementation. A description of the observed delivery of the intervention in the four intervention schools selected to participate in the qualitative data collection is provided in Table 3. Not all schools provided all components to all classes. For example, all classes were shown the DVD, but both lessons were not taught to all classes. The ‘What are germs?’ lesson was taught in three out of four observed KS1 classes, and the ‘Healthy hands, healthy school’ lesson was taught in three out of four observed KS2 classes. Results from observation checklists showed that posters were put up in classrooms and toilets in all four schools observed. Stickers were also well used, mostly distributed to pupils after they watched the DVD animation of how to wash hands, or at the end of one of the lessons. KS1 pupils at one school received a sticker as a reward after the teacher had observed them washing their hands properly. The optional components of the educational package (homework, art competition, game) were not used in the four schools observed. Only two teachers made use of the fun facts as additional information in their lessons (Table 3).

**Table 3:** Components of the intervention observed in the four intervention schools participating in the direct observation

**Component**	**Observation notes**	**Number of classes using component**		**Duration for teaching component Range, minutes**
		**KS1 N=4**	**KS2 N=4**	
DVD or CD-ROM animation	All schools used the animation. In two schools it was shown as part of whole school assemblies. In one school it was shown to separate KS1 and KS2 assemblies and combined with a demonstration of how to wash hands according to the DVD instructions, with input from pupils. At another school the DVD was shown to individual classes in combination with other intervention lessons. The instructions in the DVD were used by one KS1 teacher as a vehicle for teaching time connecting words such as “first”, “then” and “next” in a grammar lesson.	4	4	10 to 25
‘What are germs?’ lesson	At one school this lesson was only used for KS2, not KS1. The colouring germ character worksheets were used in KS1 classes. Time spent on the content of this lesson ranged from 8 minutes in a class where it was combined with the ‘Healthy hands, healthy school’ lesson, to 75 minutes in a KS2 class where the pupils were interested and engaged. Three KS2 classes designed their own germ, as suggested in the lesson plan. One KS2 class also used the germ character colouring worksheets designed for KS1.	3	4	8 to 75
‘Healthy hands, healthy school’ lesson	All KS1 classes used the glitter activity. One KS1 teacher used the glitter activity as a science experiment. The KS2 teacher who did not use this lesson explained that they used the ‘What are germs?’ lesson, and left the glitter lesson to the younger pupils. In one KS2 class the activity was scaled down due to limited time, so that only 6 pupils demonstrated the glitter activity rather than the whole class.	4	3	11 to 60
Posters	Posters were used in all schools, displayed near sinks, on bathroom walls and classroom doors. One KS1 class cut out pictures from photocopied posters and pasted them to make their own hand washing instruction pictures. Another KS1 class used the poster to review correct hand washing technique. Posters at one school were laminated to enable them to be displayed for a longer period of time.	4	4	-
Stickers	Stickers were used at all schools, sometimes distributed to all pupils after watching the animation, or provided as a reward for correct hand washing technique.	4	4	-
Fun facts	Additional background information about hand washing and germs were used in lessons by two KS2 teachers.	0	2	-

*Note*. *KS1* Key Stage 1, *KS2* Key Stage 2, The homework worksheets, art competition and game were not observed in process evaluation schools.

While data on the specific components of the intervention delivered were not systematically collected for all schools in the study, particularly among control schools, ad hoc communications recorded on log sheets supported the observations from the process evaluation schools that there was variation in the dose of the intervention delivered. For example, five intervention schools stated that they did implement the homework, but two intervention schools stated that they did not because they had no-homework policies. Staff at four intervention schools commented on their use of stickers but a head teacher at another intervention school indicated they did not use stickers for the older pupils.

### Fidelity

Communications with schools recorded in the log sheets indicated that most intervention schools delivered the DVD and lessons with fidelity, as described in the resource pack and during the telephone discussion with a HPA or University staff member. Of the 34 intervention schools that provided specific details about the intervention delivery, 33 schools stated that they showed the DVD at whole school assemblies, consistent with the instructions. Fewer details were recorded about the intervention delivery among control schools in the log sheets but feedback received in interviews with coordinating HPA and university staff indicated that fidelity was perceived to be better when the intervention was taught in intervention than in control schools.

I think some of the schools might have used it in a class here or there, but it wasn’t necessarily that they used it as well as we wanted them to in this year’s study (control schools), whereas the previous year’s study (intervention schools) many more schools used it the way we wanted them to. [ID 2, HPA staff interview]

In the four intervention schools included in the process evaluation observations, the DVD was shown to pupils in different environments. In two schools it was shown as part of whole school assemblies. In one school it was shown to separate KS1 and KS2 assemblies and combined with a demonstration of how to wash hands according to the DVD instructions, with input from pupils. At another school the DVD was shown to individual classes in combination with other intervention lessons. In these four schools, time spent on the ‘What are germs?’ lesson ranged from 8 minutes in a class where it was combined with the ‘Healthy hands, healthy school’ lesson, to 75 minutes in a KS2 class where the pupils were interested and engaged (Table 3). The lesson plan recommended all children take part in the glitter activity, but in one class a few pupils demonstrated while the rest watched.

It was for the whole class to get glittered wasn’t it? So I just scaled it down because we didn’t have much time. [ID 303, KS1 teacher interview]

### Acceptability

HPA staff reported generally positive feedback from schools that had used the resource.

Usually if they had used the (resource) pack, it was positive… I think they found it useful and also the fact that the pack had the lesson already prepared for them, so it was something else that they didn’t have to worry about. [ID 2, HPA staff interview]

Many pupils and teachers enjoyed the DVD and thought it fun, although one pupil said it was boring.

It wasn’t just like strict ‘you have to wash your hands’ , it had like a kids cartoon so you could actually pay attention and catch like the kids’ side. [ID 303, upper KS2 focus group]

Pupil A: A couple of weeks ago we watched a DVD about hand washing and…

Pupil B: Hands up for Max.

Pupil C: It was about how to wash your hands and when you should wash your hands.

Pupil A: No it was boring.

Pupil C: I thought it was actually quite interesting.

Pupil D: Yeah I liked it.

Pupil C: Most of us didn’t know how to wash our hands really properly. [ID 317, lower KS2 focus group]

While some teachers thought the cartoon was appropriate for all ages, some thought it was too young and not relevant for older pupils.

The video was quite good they liked it, for our class. It was a bit too patronising for the older ones. They (younger pupils) thought it was quite funny, when they were watching it they were like “Oh, that’s the little man from the posters”, they realised it connected so they liked the video and they liked the musical bits. [ID 317, KS1 teacher interview]

I liked the DVD, I thought it was very simple but very effective… I think it worked well with both (age groups). [ID 303, KS2 teacher interview]

The children laughed at the DVD and perhaps it wasn’t quite so appropriate for us at KS2 as it would be for other age groups. [ID 317, KS2 teacher interview]

Some pupils found the worksheets difficult, whereas some teachers adapted the lesson plan when they felt the worksheets were inappropriate. For example some teachers thought the cartoon images of germs were either too juvenile for older pupils, or too unrealistic to provide accurate information to younger pupils.

Pupil A: I always thought we found it really difficult. I think it was the first lesson we had, do you remember?

Pupil B: The quiz.

Pupil C: This germ’s called… it’s spread by…

Pupil A: Yeah it was quite hard. [ID 317, upper KS2 focus group]

I felt that the worksheets that were there to back them up we hadn’t covered the things in the lessons, the things like the different types of viruses… so they couldn’t do them. And quite a lot of them when we had to design our own germ and they looked at some of the germs that were on the sheets and were saying “Germs haven’t got legs, this is silly” and I wondered how appropriate that was for upper KS2… because you know we do work on germs anyway… and we’re teaching them that germs are very microscopic… and it sort of contradicts the way we’ve done it. [ID 317, KS2 teacher interview]

I wouldn’t do the cartoons because the children, they take it literally at that age… then the children will just… presume that all germs have skateboards and skates and have funny glasses on so I think if you’re going to show the picture it would have to be the picture what it is. [ID 317, KS1 teacher interview]

Data from observation notes about the ‘Healthy hands, healthy school’ lesson indicated that children who volunteered to demonstrate hand washing appeared “disappointed when not chosen”, and that “those not demonstrating got a little distracted as time passed”. The majority of comments related to this lesson were positive.

Of course they love glitter so that is great and it is difficult to get off… so you do have to wash your hands to get the glitter off and I think that it was a really visual way for those kids to learn about washing their hands well it’s not just visual because it’s kinaesthetic as well… I think it worked great. It’s probably the best hand washing lesson I taught. [ID 104, KS1 teacher interview]

Data from observations indicated that pupils liked receiving stickers and one teacher thought the posters and stickers made the ‘Hands up for Max!’ program better than a previous hand hygiene program.

I think this has been stronger (than School Council programs) because there has been nice visual posters to look at and it’s that gimmick… stickers to go home, it does hit home a little bit more. [ID 303, KS1 teacher interview]

Sending the resource electronically may make it easier to disseminate and use.

I think it would be useful if it could be electronic because I think then as well for us it would be easier to send out because you’re not relying on sending it out through the post, and they’ve then got an electronic copy that’s not going to get dog-eared that they can then print out easier. [ID 7, HPA staff interview]

### Sustainability

The first aspect of sustainability relates to the ongoing delivery of this educational resource in schools. Teachers thought the intervention could be incorporated into several areas of the curriculum and thereby taught on an ongoing basis.

Because we’ve still got the resources… I think we probably would voluntarily run it on a yearly basis and get involved in the PSHE (Personal, Social and Health Education). [ID 104, KS2 teacher interview]

I think it will be part of science because the topic we’re doing in science this term is healthy eating and healthy living so that I think will fit in well. [ID 317, KS1 teacher interview]

The second aspect of sustainability relates to how, and by whom, the educational resource is disseminated to schools. The HPA was seen to have a role in providing education and information related to hand washing, including via their website and their contact with schools, although some HPA staff did not see coordinating delivery of such a resource as part of their specific role.

We have infection control guidance that we send to schools, so we could also put in hand washing resources into that and certainly when we are dealing with schools in an outbreak situation, we can be guiding them to where they can find these sort of resources. [ID 1, HPA staff interview]

In terms of supporting public information and education, that is something that HPA do… I don’t think it has any relation to my day-to-day role at all. [ID 6, HPA staff interview]

The HPA may not have the capacity, alongside their existing competing priorities, to ensure that a large number of schools use the educational resource, and to be available for teachers during their limited non-teaching periods.

There is nobody else in this team, if the phone rings and it’s a school and they ask for me… all they can do is take the phone number of the person… you could have tried eight times and that was the only time they had managed to be able to get back to you because they are in and out of classes and that can be, not stressful, but there was no back-up here. [ID 2, HPA staff interview]

The length of time between schools agreeing to participate in the study and receiving the educational resource was crucial in terms of getting schools to successfully deliver the intervention. This is a likely contributor to the low proportion of control schools using the resource.

I just felt that the timing was quite crucial. So if you had a conversation in November, and you’re not phoning them until February, they are not going to remember, well it’s not going to be so keen and fresh in their mind. [ID 6, HPA staff interview]

To manage this issue it was suggested that the educational package be sent out in batches.

Instead of trying to do all the schools in one go… and get packs out to everybody within three days, and then have 100 phone calls to make within two weeks, I would probably do it in chunks so you would do ten schools one week and then start the next ten schools the week after… I think it would help getting back to the schools. [ID 2, HPA staff interview]

It was suggested that other agencies, for example healthy schools coordinators, health promotion nurses or education departments, who had more direct contact with schools, may be better placed to ensure delivery of the educational resource beyond the research setting.

I think only if it was maybe led more from an education department side, if it was put into the curriculum, I think that would… be a really good way to get it across to all the kids. Because our way we are doing it on a project basis, whereas if it was already part of what they have to do… I think that would be great. [ID 2, HPA staff interview]

I would have said within Healthy Schools type staff, you know those co-ordinators, but I know that they are not always around now, but somebody who is much more in touch with the school on a day-to-day or a month-to-month basis who has built up relationships with school members. [ID 5, University staff interview]

## Discussion

The educational resource was acceptable to schools and delivered in most intervention schools (85%) within the trial but few control schools (38%) outside the trial setting. Delivery of the intervention was also more likely to occur in schools who participated in the sub-study, where additional student absence data were collected, and university staff had more opportunities for contact with these schools. Fidelity of delivery was also reportedly better in intervention schools than control schools in the trial. These results suggest that delivery of the intervention beyond the trial is unlikely to be sustainable using this model of a centralised, non-research agency to coordinate intervention delivery. Some variations in dose were also apparent and optional intervention activities that might have helped to reinforce hand washing messages, particularly at home, were rarely used. Similar variations in fidelity and dose have been observed in other studies of health promotion interventions in school settings. A review of drug abuse prevention programs taught by teachers found considerable variability in the number of key curriculum objectives covered or the number of modules taught [4]. Teachers delivering a nutrition curriculum completed 70% of the lessons with a rating of 76% faithful to the curriculum [27]. Reach in the Trial of Activity for Adolescent Girls was high, with 91% of girls in seventh-grade and 77% of girls in eighth grade taught all of the health education lessons [6]. The level of fidelity was found to be acceptable in 76% and 64% of these lessons in the first and second year of the study, respectively [6]. Whether the dose and fidelity obtained in the current study were sufficient to bring about the level of behaviour change required to reduce infection-related absence will only be able to be inferred from the main trial results. The reach obtained in intervention schools, however, compares favourably with a review of prevention and health promotion programs for children and adolescents where few studies documented implementation levels greater than 80% [1]. Few programs are also able to achieve complete implementation in real-world settings [28].

Qualitative data from this process evaluation provide useful insights for updating the educational resource if it were to be distributed more widely. Teachers generally found the intervention useful and straightforward to deliver. The element generating the most positive reaction was the glitter activity in the ‘Healthy hands, healthy school’ lesson, because of the effectiveness of the kinaesthetic aspect in demonstrating how germs spread, and the importance of hand washing. The glitter lesson may possibly be less effective if some children are passive observers rather than being actively involved. Teachers and pupils also enjoyed the DVD animation showing how to wash hands, although there was some suggestion this may be better suited to younger audiences. The stickers and posters were also well used and received. There was some concern that the ‘What are germs?’ lesson worksheets were too difficult, even for older pupils, as insufficient background information was provided for them to be able to answer the questions. In addition, the pictures of germs that could be coloured in by KS1 pupils were considered too childish because they were presented as caricatures rather than realistic, if somewhat magnified, pictures of germs.

While all four main elements of the educational resource (DVD, lesson plans, posters, and stickers) tended to be delivered to all pupils in the four intervention schools observed, the length of time spent on each lesson varied. It is likely that the depth and breadth of information covered, and therefore the understanding reached, may be quite different for a lesson lasting 10 minutes compared to one lasting over an hour. It is also possible that the rates and duration of lesson delivery noted in this part of the process evaluation may be a result, at least in part, of the classes being observed by a researcher. The trial was designed to be pragmatic, aiming for implementation to be as close to how it would be delivered in practice [29]. Adaptations teachers make so that it is appropriate and relevant to their class may be acceptable [5] and may not result in reductions of effectiveness [30], although effectiveness of this educational intervention is yet to be established. Given differing views on resources and different abilities of pupils within year levels across schools, future versions of the resource could include a range of lesson plans and teachers could choose those appropriate for their pupils. This will require communication between program developers, who understand about the essential components of an intervention and its effects, and teachers, who understand pupils, pedagogy and school settings, so that prescription and adaptability can be combined for maximum effectiveness [4].

In addition to updating the resource itself, consideration should be given to the dissemination process as HPA staff coordinating the intervention delivery had limited capacity to follow up and respond to schools. While the HPA are seen to have a role in providing hand washing information, the intervention may be more sustainable if it were embedded as part of the curriculum by education departments, and maximising effectiveness of staff such as healthy school coordinators.

A strength of this study is the use of both quantitative and qualitative methods to measure and explore intervention reach, dose, fidelity, acceptability, and sustainability. In addition, the process evaluation was conducted and analysed by a researcher not involved in the intervention delivery and before the outcome evaluation, so interpretation of key process factors likely to affect outcomes were not influenced by prior knowledge of the outcomes [31]. A limitation is that direct observation of intervention delivery was only possible within the four intervention schools. We were also unable to elicit reasons why the intervention was not delivered among some schools, due at least in part to limitations on the capacity of HPA staff to ensure that schools were using the resource.

Hand washing is a relatively simple, inexpensive and important public health measure [32]. Teaching primary school children to wash their hands properly and encouraging regular hand washing may not only reduce infection related absenteeism but also help to habitualise this behaviour at an early age. The ‘Hands Up for Max!’ intervention was a deliberately modest educational intervention, designed to be readily integrated into the existing school curriculum, and provided to schools by a public health body whose main contact with schools traditionally occurs when there is an infectious disease outbreak. Schools are an important setting for health promotion but, as this study demonstrates, simply providing carefully designed health educational resources may not be sufficient to ensure their use. The World Health Organization Health Promoting Schools framework [33] also suggests that it is insufficient to merely introduce health into the curriculum but indicates that there also needs to be concomitant changes in the school environment and in the wider community. The ‘Hands up for Max!’ intervention had a homework element with the potential to effect change in the wider community through involving parents, but this element was optional and inconsistently used even within the trial. The intervention did not include any changes to the school environment, but these may be crucial in the case of hand washing [17]. Changes to the school curriculum and environment, plus the wider community, are only likely to be achieved if health and education authorities work together on a regular and continuing basis. This integrated working can create a shared understanding that education can improve health, and that improving health in schools can also improve educational attainment [34].

## Conclusions

The ‘Hands up for Max!’ educational intervention was well received by teachers and pupils and if results from the main trial show that the intervention is effective in reducing absenteeism, there will be grounds for introducing the intervention into more schools or making it part of the primary curriculum. However, data from this process evaluation indicate that the current mode of delivery, through the HPA, is likely to result in few schools actually using the resource. New ways of disseminating the educational resource, including embedding it in the curriculum, having electronic versions, making more use of relevant staff such as healthy schools coordinators, and supporting the dissemination of the resource with changes to the school environment will need to be considered.

## Abbreviations

HPA: Health protection agency; KS1: Key stage 1; KS2: Key stage 2; RCT: Randomised controlled trial.

## Competing interests

Rona Campbell is a Director of DECIPHer IMPACT a not-for-profit company wholly owned by the Universities of Bristol and Cardiff. The company licences use of evidence-based health promotion programmes and provides materials, training and quality assurance to support their delivery.

## Authors’ contributions

CRC was involved in design of the process evaluation, and collected, analysed and interpreted data, and drafted and revised the manuscript. ALN and SB were involved in study design, data collection, interpretation of data and revising the manuscript. EY collected, analysed and interpreted data and revised the manuscript. RC conceptualised and designed the study, interpreted data and revised the manuscript. All authors read and approved the final manuscript.

## Pre-publication history

The pre-publication history for this paper can be accessed here:

http://www.biomedcentral.com/1471-2458/13/757/prepub
